# The npj Digital Medicine Editorial Fellowship

**DOI:** 10.1038/s41746-025-02136-6

**Published:** 2025-11-03

**Authors:** Ben Li

**Affiliations:** 1 Division of Vascular Surgery, University of Toronto, Toronto, ON Canada; 2 Temerty Centre for Artificial Intelligence Research and Education in Medicine, University of Toronto, Toronto, ON Canada

**Keywords:** Health care, Medical humanities, Medical research

## Abstract

The *npj Digital Medicine* Editorial Fellowship (https://www.nature.com/npjdigitalmed/editorial-fellowship) is a year-long program that provides trainees and early career researchers with direct exposure to peer review, editorial writing, and journal operations with *npj Digital Medicine*. Since 2021, the program has graduated 4 fellows, who remain active with the journal as reviewers, editorial board members, and guest editors. As the 2024–25 Editorial Fellow, I discuss the fellowship’s structure, outcomes, and learning experiences.

## Introduction

Editorial fellowships provide an important opportunity for trainees and early career researchers to learn about the peer review process, manuscript handling, and journal operations^1,2^. This not only supports their own publishing practices but also prepares them to become robust reviewers, editorial board members, and editors^1,2^. Although several editorial fellowships exist, few have focused on transformative areas in medicine, such as artificial intelligence and digital health^3,4^. To address this gap, *npj Digital Medicine* established an Editorial Fellowship in 2021, teaching fellows how to assess and write about cutting-edge research in digital medicine^5^. The fellowship is managed by Editor-in-Chief Dr. Joseph Kvedar and Publishing Editor Dr. Tony Chen^5^. An open call for applications takes place each year and one candidate is selected to enter the program^5^. In the 2025–26 application cycle, there were 45 applications for one position. Applicants came from diverse backgrounds and geographic regions, with varied levels of training, areas of focus, and expertise. These descriptors highlight the strong and broad interest in the fellowship. This article discusses the structure and outcomes of the *npj Digital Medicine* Editorial Fellowship, along with my personal learning experiences as a recent graduate of the program.

## Fellowship structure

The 1-year fellowship is divided into three phases, each lasting 4 months^5^.

1. *Peer review phase:* The fellow writes at least 2 peer review reports per month for manuscripts submitted to *npj Digital Medicine*. The Editor-in-Chief provides direct feedback to the fellow to help them write high-quality, thoughtful, and constructive reviews.
2. *Editorial phase:* Working with 2 Editorialists and the Editor-in-Chief, the fellow writes approximately 2 editorials per month. The fellow identifies recently published work in the journal with particular importance and writes a peer-reviewed editorial about the implications of the work in non-technical language accessible to a broad audience.
3. *Operations phase:* The fellow attends weekly editorial team meetings to learn about the daily operations of the journal, including handling of manuscripts, team management, and promotional activities. With the help of the editors, the fellow identifies one or more projects they will work on to contribute to the journal’s activities, such as strengthening the peer review process, expanding the journal’s reach, or increasing overall impact.

## Fellowship outcomes

Since the fellowship was established in 2021, there have been 4 graduates (Jayson S. Marwaha, Mirja Mittermaier, Jethro C.C. Kwong, and Ben Li) and 1 current fellow (Ariel Ong)^5^. The fellows are based across a wide geographical spread, including Germany, United Kingdom, United States, and Canada^5^. They also have diverse clinical and academic interests, including machine learning in surgery, data analytics in respiratory medicine, and artificial intelligence systems in retinal disease^5^. Many of the fellows have gone on to pursue higher level academic and clinical training and remain active with *npj Digital Medicine* as reviewers, editorial board members, and guest editors^5–7^.

## Learning experiences

Fellows have consistently highlighted the positive learning experience provided by the fellowship based on publicly available personal recounts through academic blog posts^8,9^. Jayson S. Marwaha (2021-22 Fellow) explained that the fellowship allowed him to gain exposure to the highest quality work in digital medicine, collaborate with leaders in the field, and better understand the daily activities of the journal that allow it to remain impactful in a rapidly evolving field^8^. Mirja Mittermaier (2022–23 Fellow) highlighted how the fellowship strengthened her ability to write constructive peer reviews, improved her communication skills, and helped her stay at the forefront of digital health^9^.

As the 2024–25 Editorial Fellow, I peer-reviewed 10 manuscripts, published 7 editorials^10–16^, and developed a collection on transforming medical education through artificial intelligence^7^. During the peer review phase, I learned how to write constructive reviews on a broad range of digital medicine topics, from sensor-based smartphone applications to generative artificial intelligence policy frameworks. In the editorial phase, I learned how to summarize complex digital medicine topics in non-technical language and describe the implications of important papers in an easy-to-understand manner for a broad audience. During the operations phase, I learned how to identify an important topic in digital medicine and develop a collection inviting authors to submit related work to the journal^7^.

Throughout the fellowship, I had incredible support from the Editor-in-Chief, Publishing Editor, Associate Editors, and other members of the *npj Digital Medicine* community^6^. I also worked closely with other trainees including the Editorialists, Communications Fellow, and News and Views Student Editor^17,18^. This community of mentorship and support was instrumental to my learning and helped me better appreciate the diversity of perspectives in digital medicine and the importance of teamwork in maintaining a top-tier journal.

I particularly enjoyed the flexibility of the fellowship. I met one-on-one monthly with the Editor-in-Chief. During these meetings, we assessed my progress, identified areas of improvement, and established/refined goals. These meetings helped me find unique ways to contribute to the journal as a trainee. Early on, I expressed interest in strengthening my writing skills, and the Editor-in-Chief connected me with the News and Views Editors^6^. Working with them, I published 4 News and Views articles on a variety of topics that I was interested in, including commercialization of medical artificial intelligence technologies, advancing perioperative care with digital applications, and artificial intelligence innovations that have won Nobel Prizes^19–22^. The fellowship offered me the flexibility to go beyond the formal curriculum and explore my unique interests in digital medicine, which helped me develop my own voice with the journal.

Over the next year, I plan to continue working on the medical education collection that I developed during the operations phase of my fellowship (https://www.nature.com/collections/ehadbhgiji)^7^. As the guest editor of this collection, I will be handling manuscripts submitted to the collection. My role involves assessing manuscript suitability for the journal, inviting reviewers, assessing reports, and making publication recommendations to the Editor-in-Chief. These tasks represent the culmination of the knowledge and skills that I developed during the fellowship related to peer review, writing/communication, and my understanding of the journal’s audience, values, and goals.

Looking forward, I will be returning to vascular surgery residency following completion of my PhD training in machine learning. My long-term goal is to become an academic vascular surgeon focused on using advanced technologies to improve patient care. I hope to continue being involved with high-impact journals like *npj Digital Medicine*, contributing to the ongoing production and dissemination of the highest quality digital health research. I am confident that the skills I developed during the Editorial Fellowship will be invaluable in helping me reach these goals.

## Comparison with other editorial fellowships

While other well-established editorial fellowships exist, such as ones with the *New England Journal of Medicine (NEJM)*^3^ and the *Journal of the American Medical Association (JAMA)*^4^, the *npj Digital Medicine* Editorial Fellowship is unique in several ways. First, while other journals often take multiple fellows per year, *npj Digital Medicine* accepts one fellow each year^3–5^. This allows the fellow to receive dedicated attention from the journal and provides the fellow with opportunities to customize their learning based on their unique interests and skills. Second, *npj Digital Medicine* has a more focused scope than general medical journals like *NEJM* and *JAMA*^23–25^. This allows the fellow to hone their knowledge in digital medicine and develop a specialized skillset in their field of interest. Third, artificial intelligence is transforming health care, and the *npj Digital Medicine* Editorial Fellowship provides trainees and early career researchers with the technical knowledge to assess often complex studies and become academic leaders in this rapidly evolving field^26^. For these reasons, the *npj Digital Medicine* Editorial Fellowship is uniquely positioned to train the next generation of scientific leaders in digital medicine.

## Conclusions

Since 2021, the *npj Digital Medicine* Editorial Fellowship has graduated 4 fellows who remain active with the journal as reviewers, editorial board members, and guest editors. Unique aspects of the fellowship include dedicated attention from the journal with one fellow per year, flexibility to explore interests beyond the formal curriculum, and focus on transformative research in digital medicine. As the most recent graduate, I can confidently say that the fellowship has significantly strengthened my understanding of scientific publishing and prepared me well to continue my academic pursuits in artificial intelligence and digital health. With an open call for applications each year, the *npj Digital Medicine* Editorial Fellowship is well-positioned to train the next generation of scientific leaders in digital medicine and can serve as a blueprint for developing high-quality journal-based editorial fellowships or similar training programs.

## Data Availability

No datasets were generated or analysed during the current study.
